# In Situ Polymerization Synthesis of Graphdiyne Nanosheets as Electrode Material and Its Application in NMR Spectroelectrochemistry

**DOI:** 10.3390/polym15122726

**Published:** 2023-06-18

**Authors:** Siyue Zhang, Lin Yang, Xiaoping Zhang, Yuxue Chen, Yutong Zhang, Wei Sun

**Affiliations:** Key Laboratory of Laser Technology and Optoelectronic Functional Materials of Hainan Province, Key Laboratory of Functional Materials and Photoelectrochemistry of Haikou, College of Chemistry and Chemical Engineering, Hainan Normal University, Haikou 571158, China

**Keywords:** graphdiyne, in situ, NMR, spectroelectrochemistry, hydroquinone

## Abstract

In situ NMR spectroelectrochemistry is extremely powerful in studying redox reactions in real time and identifying unstable reaction intermediates. In this paper, in situ polymerization synthesis of ultrathin graphdiyne (GDY) nanosheets was realized on the surface of copper nanoflower/copper foam (nano−Cu/Cuf)-based electrode with hexakisbenzene monomers and pyridine. Palladium (Pd) nanoparticles were further deposited onto the GDY nanosheets by the constant potential method. By using this GDY composite as electrode material, a new NMR-electrochemical cell was designed for in situ NMR spectroelectrochemistry measurement. The three-electrode electrochemical system consists of a Pd/GDY/nano−Cu/Cuf electrode as the working electrode, a platinum wire as the counter electrode, and a silver/silver chloride (Ag/AgCl) wire as a quasi-reference electrode, which can be dipped into a specially constructed sample tube and adapted for convenient operation in any commercial high-field, variable-temperature FT NMR spectrometer. The application of this NMR-electrochemical cell is illustrated by monitoring the progressive oxidation of hydroquinone to benzoquinone by controlled-potential electrolysis in aqueous solution.

## 1. Introduction

In situ NMR spectroelectrochemistry has demonstrated power in the determination of unstable intermediate products and final products in electrochemical reactions [1,2,3]. Richards et al. [4] first designed the in situ liquid state electrochemistry-nuclear magnetic resonance cell (EC−NMR), which fuses NMR and electrochemistry into one. In the wake of a growing need for fast NMR structural elucidation of electrochemically generated compounds, the in situ electrochemistry-combined nuclear magnetic resonance (EC−NMR) technique has attracted increasing attention [5,6,7,8,9]. Yao et al. [6] measured macroscopic thermodynamic and kinetic properties of metal−organic frameworks (MOFs); using in situ ^1^H nuclear magnetic resonance (NMR), a mechanistic of the molecular adsorption process and kinetics in metal-organic framework UIO−66 was obtained. Albert et al. [10] tracked the anodic conversion of 2,4,6−tri-tert−butylphenol by two types of ^13^C NMR experiments: a static procedure with the electrodes directly into a rotating NMR tube, and a continuous flow procedure using electrochemical and NMR flow cells. In the first experiment, the electrode assembly is inserted directly into the NMR tube and the NMR spectra are recorded in the conventional manner after stopping the electrolysis (about 15 min). The second approach allows the continuous observation of conversion using a flow-through NMR cell, the electrolysis being maintained in an electrochemical flow reactor coupled to the NMR cell. The resolution of the ^13^C NMR spectra decreased in the static procedure by reason of magnetic field gradients in electrolysis. In the flow procedure, the resolution was comparable with that of routine spectra. Owing to the fact that the EC cell is far away from the NMR detection area in the continuous flow procedure, the interference of EC and NMR is avoided, which is beneficial to the tuning and leveling of the NMR. The disadvantages of the device lie in the long reaction time arising from the large volume of reaction solution. To solve the problem of long reaction time, Mincey et al. [11] designed a metal film−coated EC cell, which consists of a 5 mm Sb−SnO_2_−coated tube as the working electrode (WE), a platinum wire wrapped (15 turns) around a capillary tube as the counter electrode (CE), and 0.25 mm sliver wire as a quasi-reference electrode (RE). They first proposed that when the skin depth of the metal coating in the radio−frequency field is much greater than the thickness of the metal coating, the effect of EC on NMR can be reduced. Bussy et al. [12] designed a more convenient in situ EC cell, with carbon microfibers being used as WE and RE. They used the device to monitor unstable quinone imines and to elucidate their chemical structures.

In situ EC-NMR technology is extremely powerful at researching redox reactions in real time and identifying unstable reaction intermediates; however, the implementation of an EC cell near the sensitive volume of an NMR probe observably affects the quality of the NMR signal, inducing partial loss of the multiplet structures and peak overlap. Hence, Boisseau et al. [13] presented an ultrafast 2D spectroscopy coupling with EC−NMR to deal with the issue; real-time EC−2D NMR high-resolution spectra during the electrochemical reduction of 9−chloroanthracene demonstrates the feasibility of coupling in situ spectroelectrochemistry with ultrafast 2D NMR spectrum, and the approach opens a number of promising perspectives for the structural elucidation of electrochemical reactions. Zalibera et al. [1] for the first time proposed a powerful method coupling EC with ^19^F NMR in the elucidation of redox mechanisms of fluoroorganic compounds. Moreover, they display the importance of combining different spectroelectrochemical methods and quantitative analysis of the transferred charge and spin in determination of the redox mechanism. Use of EC−^19^F NMR method for in situ EC-NMR experiments is not limited to ^1^H NMR and ^13^C NMR. Grey et al. [14] used ^31^P NMR and ^19^F NMR to record the ion concentration of cation in the porous electrode during charging and discharging in real time, which revealed the energy mechanism of the supercapacitor.

Graphdiyne (GDY) is an artificial carbon allotrope containing sp and sp^2^ hybridized carbon forming a 2D framework [15]. Due to its unique nanotopological pores, two-dimensional layered conjugated frameworks, and excellent semiconducting and optical properties, GDY has displayed distinct superiorities in the fields of electrocatalysis, photocatalysis, energy storage, electrochemical sensor, etc. [16,17,18,19,20,21]. Palladium (Pd) nanoparticles are well acknowledged to possess high catalytic activity, good stability, and excellent conductivity [22,23]. So, Pd nanoparticle-modified electrodes exhibit excellent electrocatalytic effect for many chemical reactions [24,25,26]. Mao et al. [27] prepared Pd/graphdiyne oxide (GDYO) nanocomposite, which exhibited a high catalytic performance toward the reduction of 4-nitrophenol. Tong et al. [28] synthesized a well-designed Pd/graphdiyne/graphene (GDY/G) nanocomposite; its high catalytic performance also was demonstrated by the reduction reaction of 4-nitrophenol. In summary, Pd/graphdiyne nanocomposite is a new promising catalytic material which can be used in a broad range of future electrocatalytic reactions.

As a model redox system in the development of electrochemistry. hydroquinone (HQ_2_) and benzoquinone (Q) system has played a key role [29]. Prenzler et al. [30] have designed a truly practicable RF−transparent solution-NMR cell, which is applied to three-electrode/three-compartment in situ spectroelectrochemistry in a conventional 10 mm or 16 mm high-field NMR probe. The performance of this device is illustrated by monitoring the reduction of Q to QH_2_. Zhang et al. [7] designed an in situ EC−NMR cell, which consists of a PAn deposited Indium tin oxide glass electrode as the WE, a platinum wire as the counter electrode (CE), and a thin silver wire as an RE. They utilized the in situ EC-NMR cell to investigate the electro-oxidation of QH_2_ in the presence of polyaniline film as the catalyst. Zhang et al. [31] constructed a device using a three−electrode electrochemical cell with a thin gold film as the working electrode. In situ EC-NMR spectra of the electro-reduction of Q in both aqueous and non-aqueous solvents demonstrated that use of the electrochemical cell was feasible.

Herein, we designed a truly practicable three-electrode NMR-electrochemical cell using Pd/GDY/nano−Cu/Cuf electrode as the working electrode. The Pd/GDY/nano−Cu/Cuf electrode has little effect on magnetic field, so high-resolution in situ NMR spectra can be obtained. Performance of the NMR−electrochemical cell is illustrated by the oxidation of QH_2_, while the working electrode was held at a constant electrical potential. Evolution of the in situ high-resolution NMR spectra with time indicated that use of the electrochemical cell was feasible. Experimental results show that the GDY composite electrode presents a high catalytic performance toward hydroquinone electrochemical oxidation.

## 2. Experimental

### 2.1. Chemicals

All chemicals were of analytical grade and used without further treatment. Potassium hydroxide, sodium chloride, anhydrous sodium sulfate, tetrahydrofuran, tetra−n−butylammonium fluoride, ethyl acetate, N’N-dimethylformamide (DMF), acetone, pyridine, QH_2_, and Cuf were purchased from Sinopharm Chemical Reagent Co., Ltd., Shanghai, China. Furthermore, hexakis[(trimethylsilyl)ethynyl]benzene (HEB−TMS) was purchased from XFNANO, China.

### 2.2. Preparation of Pd/GDY/Nano−Cu/Cuf Working Electrode

#### 2.2.1. Preparation of Nano−Cu/Cuf

The nano−Cu/Cuf electrode was prepared by galvanostatically anodizing [32]. The copper foam was cut into a strip of 3 mm long, 1 mm wide, and 4 cm high. Cuf strips were cleaned as the working electrode, platinum wire as the counter electrode, and Ag/AgCl wire as the reference electrode. The three-electrode system was dipped into 3 M potassium hydroxide solution under the action of 8 mA·cm^−2^ for 15 min. The resulted Cuf electrode was cleaned with deionized water and dried under nitrogen flow, which was further dipped into 1 M NaBH_4_ aqueous solution for 20 min and dried in a vacuum oven for 2 h at 80 °C to obtain the nano−Cu/Cuf.

#### 2.2.2. Preparation of GDY/Nano−Cu/Cuf Electrodes

Hexakisbenzene (HEB) monomers were first obtained through deprotection of HEB−TMS. The desilication process of HEB−TMS is as follows [18]: TMS−HEB monomer (100 mg) was added to a three-necked bottle under a nitrogen atmosphere. Tetra−n−butyl ammonium fluoride (TBAF, 1 M) was immediately dissolved in tetrahydrofuran and added into the three-necked bottle at a 0 °C ice water bath. After stirring the reaction for about 10 min, the solution was transferred to the sorting funnel. Then, the appropriate amount of ethyl acetate and saturated salt water were added to the solution for extraction operation three times. Finally, the collected organic phase was dried with anhydrous NaSO_4_, then the liquid was poured into a beaker and dried in a vacuum oven at 45 °C to obtain HEB monomers. 

Freshly prepared nano−Cu/Cuf electrodes were put into a three−necked bottle with the 100 mL pyridine. Then, the deprotection HEB (20 mg) was immediately dissolved in pyridine (50 mL) and added very slowly (about 8 h) into the three-necked bottle in a glove box filled with nitrogen. The three-necked bottle was sealed in nitrogen and the reaction performed at 80 °C for 16 h away from light. After the reaction was completed, the GDY was successfully grown on the surface of nano−Cu/Cuf electrodes by in situ polymerization synthesis. The GDY/nano−Cu/Cuf electrodes were washed 3 times with DMF and acetone 3 times to remove unreacted monomers and oligomers, and then dried under vacuum at 45 °C to obtain GDY/ nano−Cu/Cuf.

#### 2.2.3. Preparation of Pd/GDY/Nano−Cu/Cuf Electrodes

**Figure 1:**
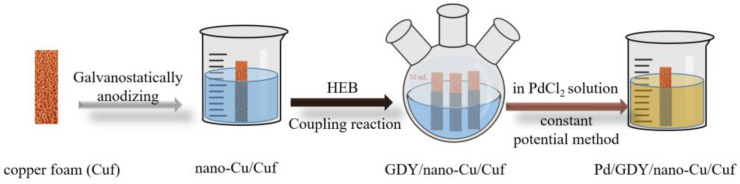
The route of preparing Pd/GDY/nano−Cu/Cuf as the working electrode.

Pd nanoparticle film was obtained via a constant potential method on the surface of GDY/nano−Cu/Cuf. The GDY/nano−Cu/Cuf electrode was dipped in PdCl_2_ (20 mM) solution at a voltage of −0.2 V with Ag/AgCl wire and Pt wire as the reference electrode and counter electrode. After lasting for 60 s, the Pd/GDY/nano−Cu/Cuf electrode was obtained. The diagrammatic flow chart of the whole synthesis procedure is shown in Figure 1.

#### 2.2.4. Design of the NMR−Electrochemical Cell

**Figure 2:**
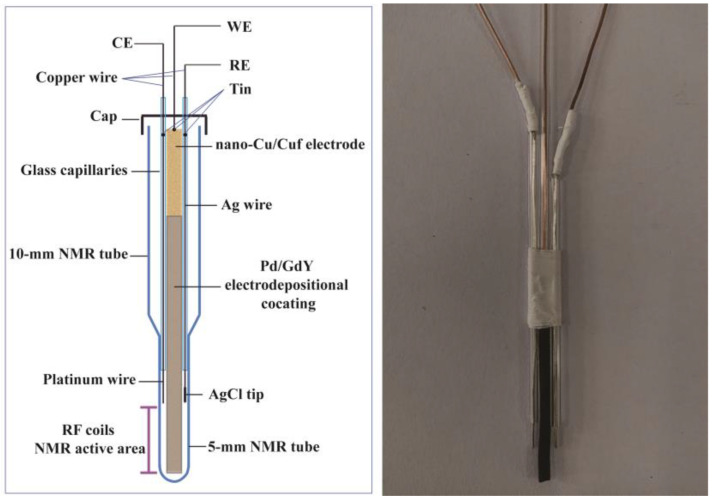
NMR-electrochemical cell designed for in situ EC-NMR.

The NMR−electrochemical cell is schematically shown in Figure 2. It consists of a Pd/GDY/nano−Cu/Cuf electrode as WE, a platinum wire as CE, and an Ag/AgCl wire as RE. To reduce the mutual interference of EC and NMR, only the part of WE located in the range of RF coils in the NMR probe. For fabrication of the CE, a copper wire and a platinum wire (0.5 mm diameter) were introduced from each side of the glass capillary. The contact between the copper wire and the platinum wire was made by a tin weld. RE was fabricated using the same method. RE and CE arranged together with the WE to avoid short circuit. The three electrodes were connected to the electrochemical workstation by copper wires and a 2 m long cable. The electrode system of all three electrodes was inserted in the NMR tube. When the in situ experiment were performed, a voltage was applied to the electrode system, and NMR monitoring was performed while the electrochemical reaction was taking place.

### 2.3. Measurements

QH_2_ (0.1 M) was dissolved in water with LiClO_4_ (1 M) as the supporting electrolyte. Electrochemical experiments were conducted with a CHI 660 E electrochemical workstation (Shanghai CH Instrument Co. Ltd., Shanghai, China). One-dimensional ^1^H NMR spectra were recorded by a Avance III HD 600 MHz NMR instrument (Bruker, Karlsruhe, Germany) at 298 K. Careful probe tuning, shimming of the magnetic field homogeneity, and 90° pulse calibration were performed before the test. The chemical shift of ^1^H NMR spectra was calibrated by the signal of the 3−(trimethylsilyl) −1−propanesulfonic acid sodium salt. The spectral width was set to 10 ppm. Each spectrum was recorded with a single scan and an experiment time of 2 s, consisting of 1 s delay time and 1 s acquisition time. The morphology of the modified nanoelectrodes was obtained on a JSM−7100F scanning electron microscope (SEM, Japan Electron Company, Tokyo, Japan). FT−IR spectrum of GDY were obtained on a Nicolet 6700 Fourier infrared spectrum instrument (Thermo Fisher Scientific Inc., Waltham, MA, USA). The sample preparation process was as follows: copper sheet was put into a three-necked bottle with 100 mL pyridine. Then, the deprotection HEB (20 mg) was immediately dissolved in pyridine (50 mL) and added very slowly (about 8 h) into a three-necked bottle in a glove box filled with nitrogen. The three-necked bottle was sealed in nitrogen and the reaction was performed at 80 °C for 16 h away from light. After the reaction was completed, the GDY was successfully grown on the surface of the Cu sheet by in situ polymerization synthesis. The GDY/Cu sheet was washed 3 times with DMF and acetone 3 times to remove unreacted monomers and oligomers, and then dried under vacuum at 45 °C to obtain the GDY/ Cu sheet. We scraped with a spoon to obtain the GDY powder. X-ray photoelectron spectroscopy of graphdiyne film was obtained on a ESCALAB 250Xi X-ray photoelectron spectrometer (XPS, Thermo Fisher Company, Waltham, MA, USA). Raman spectra of GDY were obtained on a LabRAM HR Raman spectrometer (Horiba company, Kyoto, Japan). All of the samples were measured by in situ EC−NMR experiments five times and the average data were taken. The active area of Cuf was 0.6 cm^2^ (0.3 cm × 2 cm).

## 3. Results and Discussion

**Figure 3:**
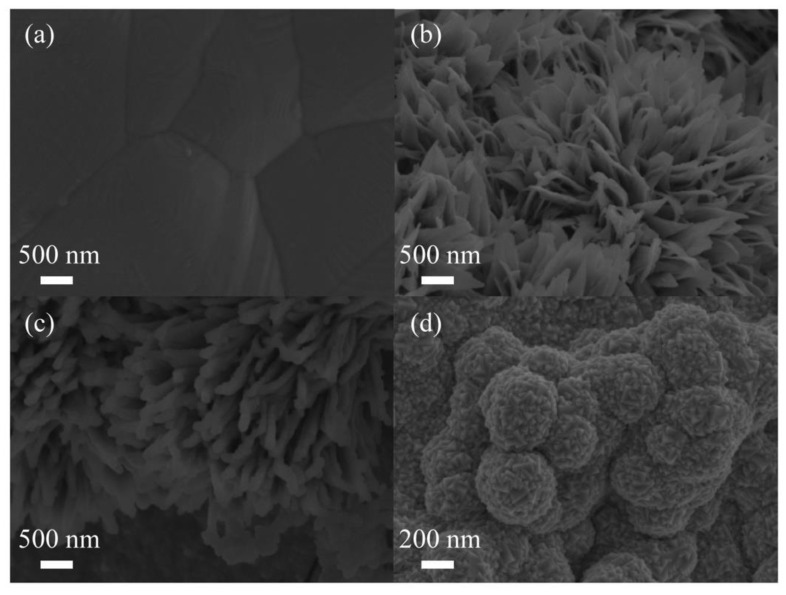
SEM images of Cuf (**a**), nano-Cu/Cuf (**b**), GDY/nano−Cu/Cuf (**c**), and Pd/GDY/ nano−Cu/Cuf (**d**).

**Figure 4:**
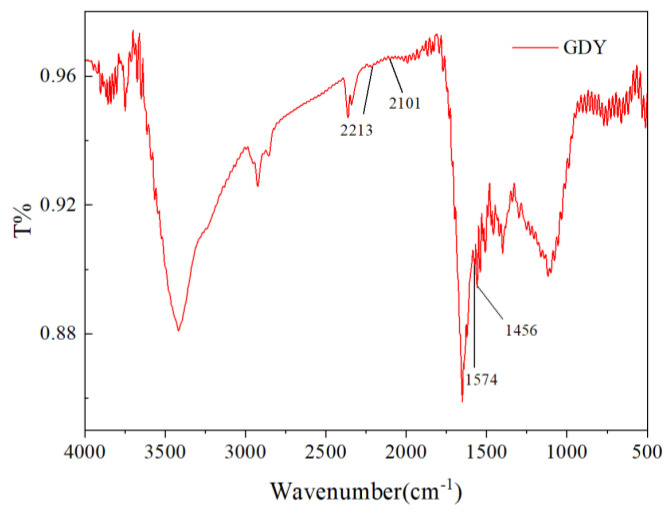
FT−IR spectrum of GDY.

The morphologies of the Cuf, nano−Cu/Cuf, GDY/ nano−Cu/Cuf, and Pd/GDY/ nano−Cu/Cuf electrodes are shown in Figure 3. As can be seen in Figure 3a, b, the morphology of Cuf changes from smooth and flat to a homogeneous nanoflower shape after electrochemical treatment. Cuf nanoflower petals appeared with width ranging from 200 nm to 300 nm and lengths of about 700 nm. Moreover, as a base material, Cuf possesses high conductivity and a unique three-dimensional porous structure. The morphology of nano−Cu/Cuf would be helpful to the flow of electrolytes and give a larger surface area. The SEM image of Figure 3c shows that the surface of nano−Cu/Cuf is covered by layers of GDY. The morphology of Pd/GDY/nano−Cu/Cuf is shown in Figure 3d. It is seen that the nanogranular of Pd tightly encapsulates the surface of GDY/nano−Cu/Cu. Due to the Pd film’s small particle size and large specific surface areas, the modified electrodes would have a high catalytic activity for QH_2_ oxidation. Figure 4 shows the Fourier transform infrared spectroscopy (FT−IR) spectrum of as-grown GDY film on Cuf. The bands located at 1456 cm^−1^ and 1574 cm^−1^ are assigned to the skeletal vibrations of aromatic ring, and the wide band of 2101 to 2213 cm^−1^ is the typical C≡C stretching vibration. The results of FT−IR spectrum are in good agreement with the reported results of GDY [33]. 

**Figure 5:**
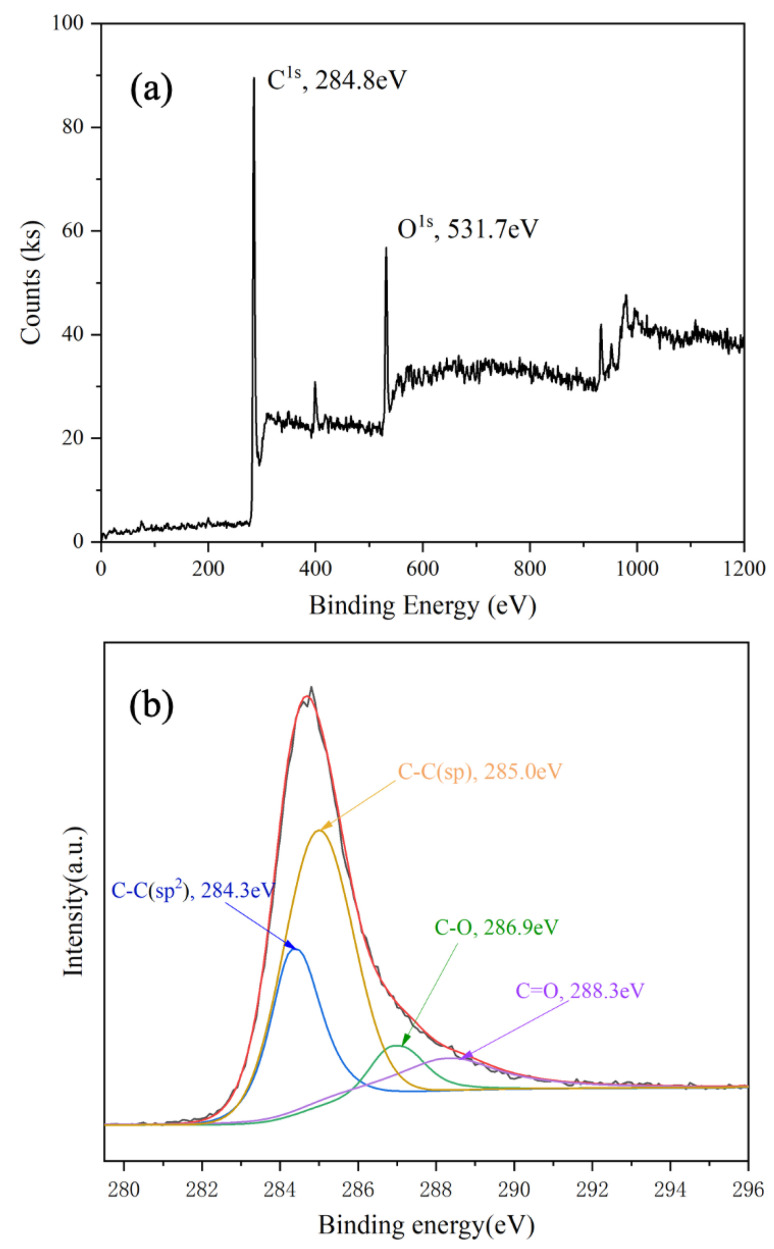
XPS spectra of graphdiyne film: (**a**) survey scan, (**b**) narrow scan for element C.

**Figure 6:**
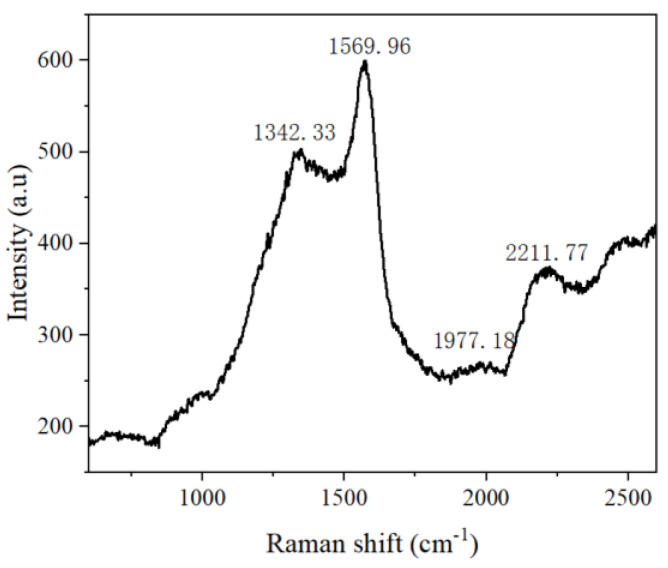
Raman spectra of GDY.

Figure 5 shows identical binding energies for the C 1 s orbital. The presence of an O 1 s peak at 5 shows the XPS spectrum of C 1 s. The C 1 s peak at 284.8 eV in Figure 5a shows that 31.7 eV is due to the absorption of air in graphdiyne. As shown in Figure 5b, four fitting peaks of 284.3, 285.0, 286.9, and 288.3 eV can be assigned to C−C (sp^2^), C−C (sp), C−O, and C=O, respectively. C−O and C=O occurs because GDY absorbs oxygen when the sample is exposed to air. The area ratio of C−C (sp)/ C−C (sp^2^) is 2, which confirms that the benzene rings link with others by diine in as-prepared GDY. GDY can be qualitatively determined according to the ratio of C−C (sp) and C−C (sp^2^), and oxygen content in XPS spectra. The result of the XPS spectrum is in good agreement with the reported results of GDY [15]. Moreover, Raman spectroscopy was used to evaluate the quality and uniformity of GDY on the surface of copper nanoflower. As shown in Figure 6, the Raman spectrum of GDY has four typical peaks, among which the peak at 1350.2 cm^−1^ corresponds to the respiratory vibration of the benzene ring, which can also be called the D band of GDY. The pronounced D band is a disordered band associated with structural defects, amorphous carbon, or edges that can break the symmetry and selection rule [33]. The peak at 1569.9 cm^−1^ corresponds to the first−order stretching vibration sp^2^ carbon domains in aromatic rings, which is the G band of GDY, and the peak at 2211.7 cm^−1^ belongs to the stretching vibration of the conjugated diyne bond [34]. The results of the SEM, FT−IR, XPS, and Raman spectrum analysis indicate that the GDY nanosheets were successfully realized on the surface of the nano−Cu/Cuf−based electrode with HEB monomers and pyridine. 

**Figure 7:**
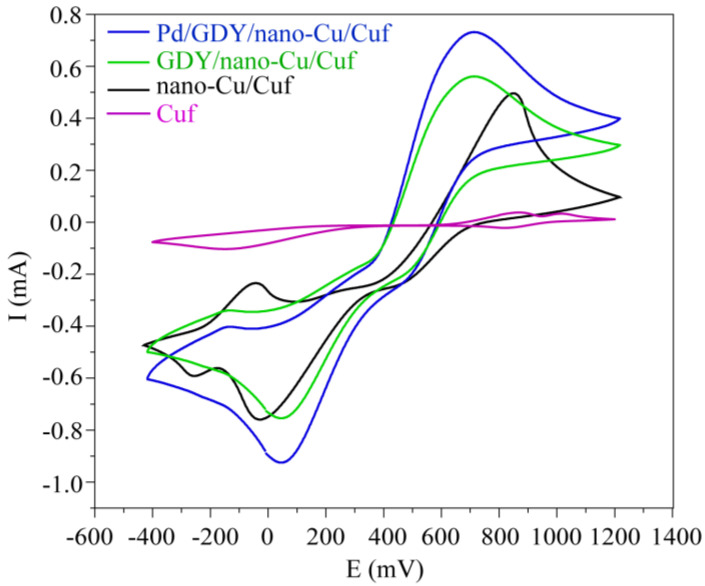
Cyclic voltammograms of QH_2_ using Cuf electrode, nano−Cu/Cuf electrode, GDY/ nano−Cu/Cuf electrode, and Pd/GDY/ nano−Cu/Cuf electrode in aqueous solution (vs. Ag/AgCl, scan rate: 10 mV s^−^¹).

To investigate the electrocatalytic behavior of QH_2_ at various electrodes, cyclic voltammograms were obtained in aqueous solution as shown in Figure 7. The oxidation of QH_2_ is a two−electron transfer process; the oxidation peaks arises at about 650 mV at Pd/GDY/nano−Cu/Cuf electrode. Compared to the results for the Cuf electrode, the redox peak currents at nano−Cu/Cuf electrode and GDY/nano−Cu/Cuf electrode were increased in sequence with the peak−to−peak separation value decreasing. The trend signifies that the reversibility of the electrode reaction improved. It is noteworthy that the redox peak currents at Pd/GDY/nano−Cu/Cuf electrode were further increased. In addition, compared to the situation for the Cuf electrodes, the redox peak current at the Pd/GDY/nano−Cu/Cuf electrode was much higher. The result indicates that the Pd/GDY/nano−Cu/Cuf electrode would present the highest catalytic performance toward QH_2_ electrochemical oxidation.

**Figure 8:**
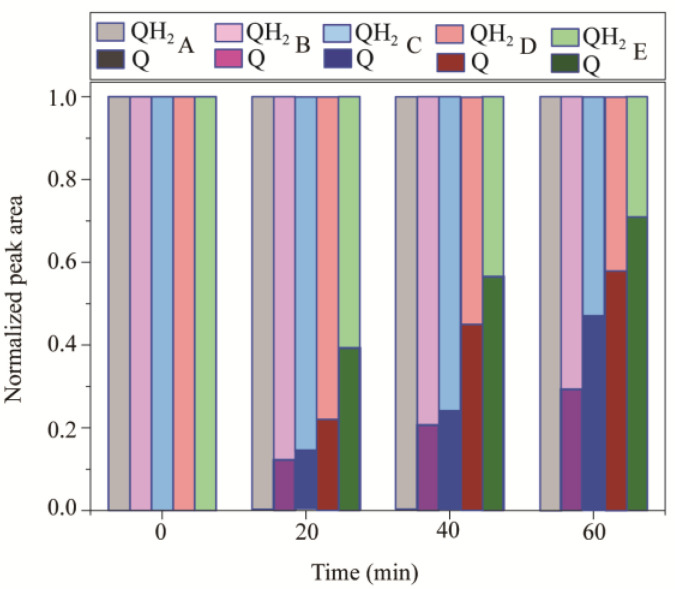
Normalized peak areas of the ^1^H NMR spectra for the hydroquinone oxidation during electrolysis process in aqueous solution at 650 mV with different electrodes (A, B, C, D, and E). These electrodes include electrodeless, Cuf, nano−Cu/Cuf, GDY/nano−Cu/Cuf, and Pd/GDY/nano−Cu/Cuf electrodes, which are labeled as A, B, C, D, and E, respectively.

To validate the performance of the NMR-electrochemical cell, in situ EC−NMR experiments of QH_2_ oxidation were executed in aqueous solution, while the working electrode was held at 650 mV. Figure 8 shows the quantitative analysis of the oxidized products over the electrolysis at various electrodes (electrodeless, Cuf, nano−Cu/Cuf, GDY/nano−Cu/Cuf, and Pd/GDY/nano−Cu/Cuf electrodes). Figure S1 shows the in situ ^1^H NMR spectra of QH_2_ at different WE (electrodeless, Cuf, nano−Cu/Cuf, GDY/nano−Cu/Cuf and Pd/GDY/nano−Cu/Cuf electrodes; these electrodes are labeled as A, B, C, D, and E, respectively) acquired at (a) 0 min, (b) 20 min, (c) 40 min, and (d) 60 min electrolysis in aqueous solution. As shown in Figure 8 and Figure S1, when we took the ^1^H NMR spectra of QH_2_ solution without electrode, only one signal at 6.62 ppm appeared and no product could be observed. Nevertheless, a new signal at 6.90 ppm arising from the protons of Q appears and increases with the electrolysis when B, C, D, and E are used as WE, respectively. At each time point, we discover that the normalized peak areas of ^1^H NMR spectra for Q from the biggest to the smallest are at E electrode, D electrode, C electrode, and B electrode. This tendency is consistent with the voltametric behavior of various electrodes in aqueous solution (Figure 7). It is observed that the Pd/GDY/nano−Cu/Cuf electrode would present highest catalytic performance toward QH_2_ electrochemical oxidation. However, the concrete value may vary from case to case because the reaction rate would be influenced by many factors, such as the thickness of GDY film, dissolved dioxygen, diffusion rate of oxidation and reduction products, electrode gap, and so on. We eliminated the effects of these factors by controlling for variables and averaging the testing values of repeated measurement. For example, all of the samples were measured by in situ EC−NMR experiments five times and the average data were taken; the active area of every Cuf was 0.6 cm^2^ (0.3 cm × 2 cm), and the electrode spacing of the three tiny electrodes was fixed using capillary glass tubes. Meanwhile, due to the fact that the NMR−electrochemical cell is micro, the diffusion rate of oxidation and reduction products matches the NMR sampling time. Moreover, by monitoring the real−time interconversion of QH_2_/Q, we can discover online the changes in reactant and reaction product. From the experimental results, it can be concluded that the use of the electrochemical cell was feasible. Furthermore, the new hyphenated technique of NMR spectroelectrochemistry provides a good approach to evaluate the electro-catalytic capacity of catalyst.

## 4. Conclusions

In summary, we designed a new NMR−electrochemical cell using Pd/GDY/nano−Cu/Cuf electrode as the working electrode. GDY modified electrode was synthesized in situ on the nano−Cu/Cuf−based electrode by a cross−coupling polymerization reaction. In situ NMR spectroelectrochemical experiments on QH_2_ oxidation were performed to illustrate the performance of the NMR−electrochemical cell. According to the experiment, the following conclusions can be proposed: 1. It is found that the fabricated three−electrode electrochemical cell has a minimal effect on magnetic field and the use of the electrochemical cell was feasible. 2. The Pd/GDY composite electrode presents a high catalytic performance toward QH_2_ electrochemical oxidation. 3. In situ NMR spectroelectrochemical technology holds potential to trace the reaction mechanism of substances, to evaluate the electrocatalysis performance of catalysts, and to detect unstable intermediates.

## Data Availability

Data is unavailable due to privacy.

## Supplementary Materials

The following supporting information can be downloaded at: https://www.mdpi.com/article/10.3390/polym15122726/s1, Figure S1: In situ ^1^H NMR spectra of hydroquinone at various electrodes (A, B, C, D, and E) acquired at (a) 0 min, (b) 20 min, (c) 40min, and (d) 60 min electrolysis in aqueous solution. These electrodes include electrodeless, Cuf, nano−Cu/Cuf, GDY/nano−Cu/Cuf, and Pd/GDY/nano−Cu/Cuf electrodes, and are labeled as A, B, C, D, and E, respectively.
