# Supplementary material for: In Situ Polymerization Synthesis of Graphdiyne Nanosheets as Electrode Material and Its Application in NMR Spectroelectrochemistry
Source: Polymers (Basel). 2023 Jun 18;15(12):2726. doi: 10.3390/polym15122726 (PMC10301388; doi:10.3390/polym15122726)
Supplement: Supplementary file 1 [file polymers-15-02726-s001.zip › polymers-2294012-supplementary.pdf]

Supplementary Information for In situ polymerization synthesis of graphdiyne nanosheets as electrode material and its application in NMR spectroelectrochemistry

Siyue Zhang, Lin Yang, Xiaoping Zhang *, Yuxue Chen, Yutong Zhang, Wei Sun *

Key Laboratory of Laser Technology and Optoelectronic Functional Materials of Hainan Province, Key Laboratory of Functional Materials and Photoelectrochemistry of Haikou, College of Chemistry and Chemical Engineering, Hainan Normal University, Haikou 571158, China

* Correspondence: 070911@hainnu.edu.cn (X.Z.); sunwei@hainnu.edu.cn (W.S.) Tel.: 13003904760 (X.Z.)

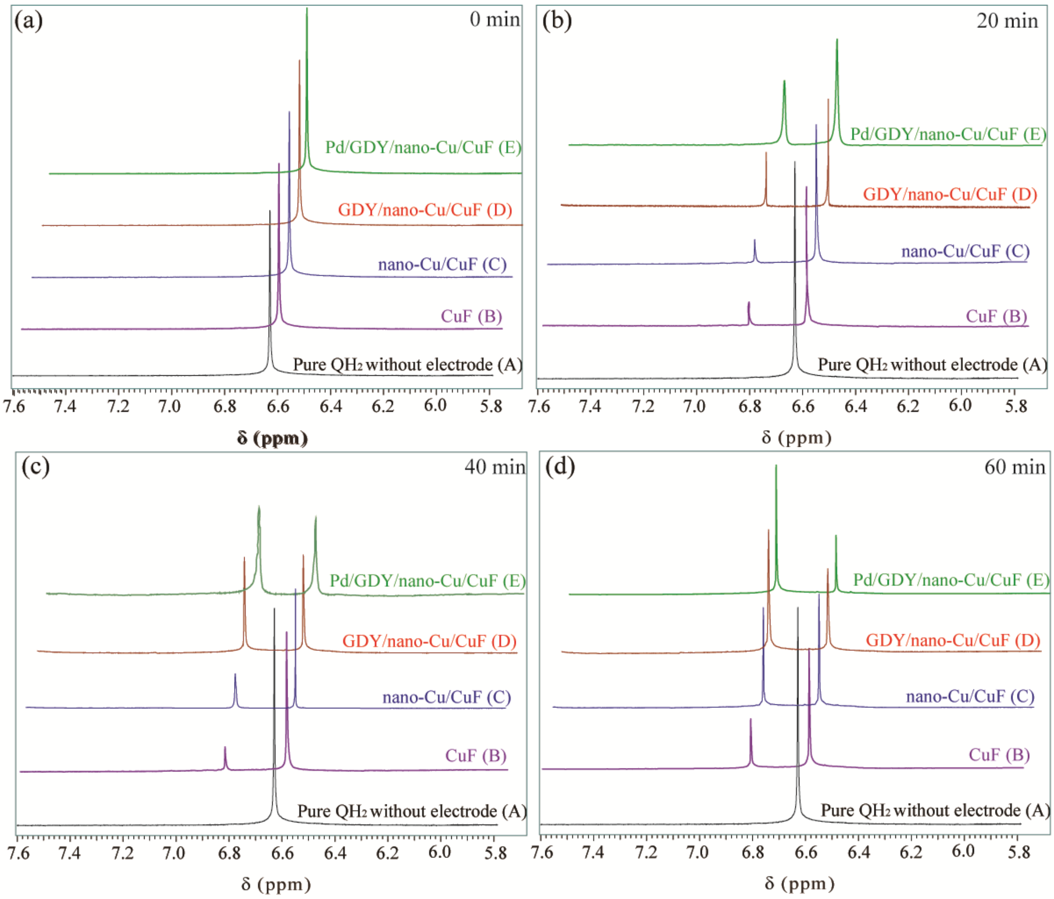

Figure S1. in situ ^1H NMR spectra of hydroquinone at various electrode (A, B, C, D, and E) acquired at (a) 0 min, (b) 20 min, (c) 40min, (d)60 min electrolysis in aqueous solution. These electrodes incloud electrodeless, Cuf, nano-Cu/Cuf, GDY/nano-Cu/Cuf and Pd/GDY/nano-Cu/Cuf electrodes, are labeled as A, B, C, D, and E respectively.
